# Change of Virchow-Robin spaces in idiopathic normal pressure hydrocephalus and its pathogenetic significance

**DOI:** 10.1186/2045-8118-12-S1-P20

**Published:** 2015-09-18

**Authors:** Masatssune Ishikawa, Shigeki Yamada, Kazuo Yamamoto

**Affiliations:** 1 Rakuwakai Otowa Hospital, Japan

## Introduction

Idiopathic normal pressure hydrocephalus (iNPH) is a disorder of aged adults, but it pathogenesis is still unknown. Recently, Virchow-Robin Spaces (VRS) are interested in anatomical structure for interaction between interstitial fluid (ISF) and cerebrospinal fluid (CSF). VRS are known to observe mainly in the basal ganglia and the subcortical white matter and they increase in number with aging. In the present study, we investigated morphological features of VRS in the brain in the aged healthy adults and patients with probable iNPH.

## Methods

Using 3-T MRI and constructive interference in steady state (CISS), 10 healthy aged adults and 30 patients with probable iNPH were examined on morphological features of VRS in the basal ganglia and subcortical white matter.

## Results

VRS in the basal ganglia were curved, irregularly sized and shaped, and communicated with the cerebrospinal fluid in the subarachnoid space; they contained perforating arteries. VRS in the white matter were straight, smooth, homogeneously sized and shaped, and did not penetrate the cortex. Arteries were not seen in VRS of the white matter. White matter VRS were sparse in patients with iNPH. Postoperatively after shunt surgery, VRS in the white matter were mildly decreased in diameter, but not in number. No significant changes were noted in basal ganglia VRS.

## Conclusions

Morphological features of VRS in the basal ganglia and white matter were different. VRS in the basal ganglia were seen as genuine perivascular spaces; while neither communication with subarachnoid spaces nor arteries were seen in white matter VRS, even by 3D-CISS sequences and high-resolution magnetic resonance angiography on 3T-MRI. White matter VRS were sparse in patients with iNPH and they were mildly decreased in diameter, but did not change in number after surgery. They may be due to dilated ISF spaces or secondary changes of amyloid deposition in the leptomeningeal arteries. or both. Further studies are necessary to elucidate the functional role of VRS in normal subjects and patients with iNPH.
